# Dignity Therapy Led by Nurses or Chaplains for Elderly Cancer Palliative Care Outpatients: Protocol for a Randomized Controlled Trial

**DOI:** 10.2196/12213

**Published:** 2019-04-17

**Authors:** Sheri Kittelson, Lisa Scarton, Paige Barker, Joshua Hauser, Sean O'Mahony, Michael Rabow, Marvin Delgado Guay, Tammie E Quest, Linda Emanuel, George Fitchett, George Handzo, Yingewi Yao, Harvey Max Chochinov, Diana Wilkie

**Affiliations:** 1 Center for Palliative Care Research and Education Department of Medicine University of Florida Gainesville, FL United States; 2 Center for Palliative Care Research and Education College of Nursing University of Florida Gainesville, FL United States; 3 Buehler Center on Aging, Health and Society Palliative Care Northwestern Feinberg School of Medicine Chicago, IL United States; 4 Palliative Medicine Rush University Medical Center Chicago, IL United States; 5 Helen Diller Family Comprehensive Cancer Center Center for Educaiton in Palliative Care University of California, San Francisco San Francisco, CA United States; 6 Department of Palliative, Rehabilitation, and Integrative Medicine The University of Texas MD Anderson Cancer Center University of Texas, MD Anderson Houson, TX United States; 7 Buehler Center on Aging, Heatlh and Society Northwestern Feinberg School of Medicine Chicago, IL United States; 8 Department of Religion, Health and Human Values Rush University Medical Center Chicago, IL United States; 9 Health Services Research & Quality HealthCare Chaplaincy Network, Caring for the Human Spirit TM New York, NY United States; 10 Research Institute of Oncology and Hematology Cancer Care Manitoba University of Manitoba Winnipeg, MB Canada

**Keywords:** palliative care, cancer, elderly, religion, therapy

## Abstract

**Background:**

Our goal is to improve psychosocial and spiritual care outcomes for elderly patients with cancer by optimizing an intervention focused on dignity conservation tasks such as settling relationships, sharing words of love, and preparing a legacy document. These tasks are central needs for elderly patients with cancer. Dignity therapy (DT) has clear feasibility but inconsistent efficacy. DT could be led by nurses or chaplains, the 2 disciplines within palliative care that may be most available to provide this intervention; however, it remains unclear how best it can work in real-life settings.

**Objective:**

We propose a randomized clinical trial whose aims are to (1) compare groups receiving usual palliative care for elderly patients with cancer or usual palliative care with DT for effects on (a) *patient outcomes* (dignity impact, existential tasks, and cancer prognosis awareness); and (b) *processes* of delivering palliative spiritual care services (satisfaction and unmet spiritual needs); and (2) explore the influence of physical symptoms and spiritual distress on the outcome effects (dignity impact and existential tasks) of usual palliative care and nurse- or chaplain-led DT. We hypothesize that, controlling for pretest scores, each of the DT groups will have higher scores on the dignity impact and existential task measures than the usual care group; each of the DT groups will have better peaceful awareness and treatment preference more consistent with their cancer prognosis than the usual care group. We also hypothesize that physical symptoms and spiritual distress will significantly affect intervention effects.

**Methods:**

We are conducting a 3-arm, pre- and posttest, randomized, controlled 4-step, stepped-wedge design to compare the effects of usual outpatient palliative care and usual outpatient palliative care along with either nurse- or chaplain-led DT on patient outcomes (dignity impact, existential tasks, and cancer prognosis awareness). We will include 560 elderly patients with cancer from 6 outpatient palliative care services across the United States. Using multilevel analysis with site, provider (nurse, chaplain), and time (step) included in the model, we will compare usual care and DT groups for effects on patient outcomes and spiritual care processes and determine the moderating effects of physical symptoms and spiritual distress.

**Results:**

The funding was obtained in 2016, with participant enrollment starting in 2017. Results are expected in 2021.

**Conclusions:**

This rigorous trial of DT will constitute a landmark step in palliative care and spiritual health services research for elderly cancer patients.

**Trial Registration:**

ClinicalTrials.gov NCT03209440; https://clinicaltrials.gov/ct2/show/NCT03209440

**International Registered Report Identifier (IRRID):**

DERR1-10.2196/12213

## Introduction

Our long-term goal is to improve spiritual care outcomes for elderly patients with cancer. We will use a spiritual intervention, dignity therapy (DT), to help these patients maintain pride, find spiritual comfort, enhance continuity of self, and ultimately explore meaning in the context of their life-threatening illness. “Dignity Therapy, a novel, brief psychotherapy, provides patients with life threatening and life limiting illnesses an opportunity to speak about things that matter most to them. These recorded conversations form the basis of a generativity document, which patients can bequeath to individuals of their choosing” [1]. However, DT has not been viewed as a spiritual intervention or studied with chaplains as the interventionist. Our thesis is that DT will systematize spiritual care processes and improve patient outcomes (spiritual and cancer prognosis awareness). The spiritual outcomes are important because people with advanced illness report that *b* *eing at peace with God is as important as freedom from pain* [2]. Spiritual concerns are issues for 86% of patients with advanced cancer [3]. Unfortunately, little research guides interventions for spiritual care. We will address this gap by testing efficacy of DT in a rigorous, multisite, randomized controlled trial (RCT) [4].

Previous studies of DT demonstrated clear feasibility but inconsistent efficacy of DT with virtually no evidence of its mechanism of action. Specifically, the 12 studies of DT (8 uncontrolled feasibility and 4 mostly small sample efficacy RCTs) show DT to be an important intervention when delivered by nurses and mental health professionals. Effects on patients’ distressing physical or emotional symptoms of life-threatening illness have been inconsistent. Taking a spiritual perspective for reanalysis of data from the 1 large RCT [1], we found that compared with usual care, patients who received DT reported significantly higher dignity impact ratings [5], which is consistent with the DT focus on meaning making, preparation for death, and life-completion tasks. Evidence from our pilot study also suggests that awareness of cancer prognosis outcomes and will-to-live is facilitated by DT [6]. Of the possible explanations for the lack of DT effect on physical symptoms, 1 could be that symptoms only moderate the DT effect, so conceptualizing symptoms as the relevant outcome is mismatched to the operative DT elements. It is also possible that spiritual distress could moderate the DT effect on patients’ sense of meaning and purpose [1], which is an important part of dignity impact.

For this study, we selected 2 disciplines of the multidisciplinary palliative care team to focus on spiritual concerns—nurses and chaplains. A nurse-led or chaplain-led DT study of patients receiving outpatient palliative care is needed to determine the efficacy of DT on key spiritual-related patient outcomes (dignity impact, existential tasks, and cancer prognosis awareness) and explore possible moderators (physical symptoms and spiritual distress) of DT’s effects on patient outcomes.

We propose a pre- and posttest, RCT with a 4-step (10 months per step), stepped-wedge design [4] to compare effects of *usual outpatient palliative care (usual care)* and usual care along with either nurse-led or chaplain-led DT on patient outcomes (*dignity impact* *, existential tasks* [preparation for death and life completion], *and cancer prognosis awareness* [peaceful awareness and treatment preferences]). We will assign 6 outpatient palliative care sites to usual care during the first step, and randomly assign 2 sites per step to begin and continue DT led by either a nurse or a chaplain during each of the next 3 steps. During the usual care steps, 280 patients will complete pretest measures (patient outcomes, covariates *[* *physical symptoms* and *spiritual distress]*, and *satisfaction* with palliative spiritual care services), receive usual palliative care, and complete posttest measures (patient outcomes, covariates, and satisfaction). During the experimental steps as part of routine palliative care service delivery, 280 patients will complete pretest measures, receive nurse-led or chaplain-led DT, and complete posttest measures. Using a mixed multilevel analysis with site, provider (nurse and chaplain) and time (step) included in the model, we will compare the usual care and each of the DT groups for effects on *dignity impact,* *existential tasks, and cancer prognosis awareness* and explore the moderating effects of physical symptoms and spiritual distress. We will also determine the effect of usual care and DT on the patient’s satisfaction with palliative spiritual care services and the patient’s unmet spiritual needs.

### Specific Aims

#### Aim 1

We aim to compare usual care and usual care with nurse-led or chaplain-led DT groups for effects on *(1)* *patient outcomes* (dignity impact and existential tasks [preparation for death and life completion], cancer prognosis [peaceful awareness and treatment preferences]). We *hypothesize* that, controlling for pretest scores, each of the DT groups will have higher scores on the dignity impact (primary outcome) and existential tasks (secondary outcome) measures than the usual care group. In addition, patients in each of the DT groups will report better peaceful awareness and treatment preferences more consistent with their cancer prognosis (secondary outcomes) than the usual care group; and (2) *processes* of delivering palliative spiritual care services (satisfaction and unmet spiritual needs; and secondary outcomes). We *hypothesize* that each of the DT groups will show increased patient satisfaction with spiritual care services and fewer unmet spiritual needs compared with the usual care group.

#### Aim 2

We aim to explore the influence of physical symptoms and spiritual distress on the dignity impact and existential tasks effects of usual palliative care and nurse-led or chaplain-led DT. We *hypothesize* that physical symptoms and spiritual distress will significantly affect intervention effects. *This rigorous trial of DT will constitute a landmark step in gero-oncology palliative care and spiritual health services research.*

### Research Strategy and Significance

The proposed palliative care research is responsive to PA-13-354/NOT-CA-14-016, *Advancing the Science of Geriatric Palliative Care* for Cancer Patients. It is significant for multiple reasons: it breaks new ground in inquiry regarding spiritual care as a part of patient-centered care for elders with serious illness before they get morbidly ill. The study uses an empirically established intervention (DT), opening chaplaincy in palliative care to rigorous health services research. The study advances palliative care research by leveraging a network of collaborators in a recently formed network for large data, and by making use of a relatively novel method, stepped-wedge design that typically reduces sample size needs and makes recruitment more feasible, especially for those who are frail and stressed by their cancer illness and treatments.

### Dignity Therapy: An Effective Intervention in Need of More Study

DT has its conceptual origins in Butler’s Life Review [7], which he developed as an antidote to depression in elders and understood as part of a life-cycle task. Both interventions are conceptualized as psychosocial and multidimensional for patient-centered care. But little is known about how DT works and if it can and should be used clinically.

Although DT has been established in RCTs to be beneficial to patients among multiple disease groups in multiple ways, the question of its efficacy when *administered* in a real-life care setting is not established. Pragmatically, within palliative care teams, it is most likely that nurses or chaplains could include DT as part of their routine work, and we, therefore, have designed a trial to assess the efficacy of DT delivered by nurses or chaplains. Considerations for either discipline include the following facts. In a prior RCT [1], a research nurse administered the DT with significant dignity impact findings. Furthermore, the nursing discipline’s focus on holistic care inclusive of spirituality makes this discipline a strong candidate. Although the ratio of nurses to patients is suitable, nurses already have a heavy flow of work that may be sidetracked by DT unless carefully scheduled. In addition, recent work indicates that a taxonomy of chaplaincy activities reasonably aligns with DT components [8,9] and recent findings [10] also indicate that clinicians from nursing and medicine look to board certified chaplains (BCCs) as the professionals with the expertise to provide spiritual care. Chaplain-to-patient ratios and their assignments are not currently suitable for routine offering of DT, but chaplains might be more interested in DT than nurses due to alignment of DT with chaplain tasks. If our findings show that DT led by nurses or chaplains has outcomes better than usual care, in the future, DT could be implemented in many places by redeploying current nursing or chaplain resources, based on their availability. Fortunately, *six palliative care outpatient services* are committed to test the efficacy of DT led by either nurses or chaplains and thereby provide evidence about the effects of DT compared with usual care in the context of outpatient palliative care of elderly cancer patients.

As palliative care starts earlier in the course of medical therapy and *geriatric oncology* care continues to seek interventions for ambulatory elders, we have asked if DT can be successfully administered in outpatient settings. The findings from a pilot study conducted in a colorectal cancer clinic indicate  *feasibility* in an outpatient setting [6]. This pilot study provides guidance about where DT should be done and when in the sequence of the patient’s clinical encounters. Our experience with this study also helped us plan who should transcribe the interview, how to return the legacy document, and how long an interval between transcription and delivery is suitable. None of these decisions are established for clinical practice, but the proposed study will provide evidence from 6 sites to better inform future clinical practice implementation of DT, including the impact on the flow of the team’s work and DT’s impact on illness acceptance and cancer care goals. Successful completion of the proposed study is likely to have a significant and sustained impact on gero-oncology care in the future.

**Figure 1:**
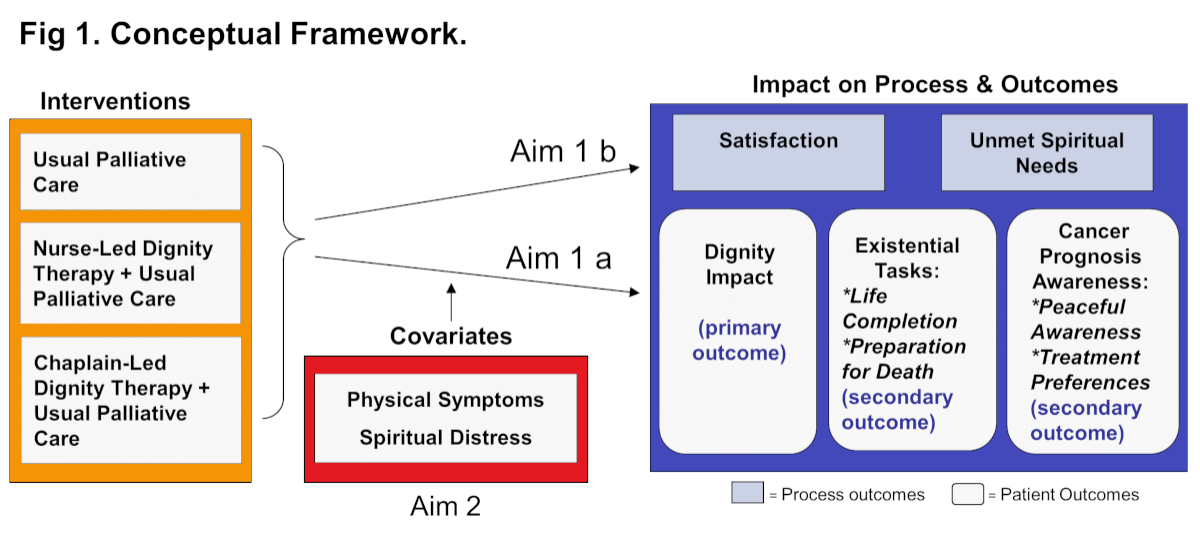
DT_Fig 1_Conceptual Framework.png.

### Spiritual Interventions Are Important to Patients but Understudied

Many  *elders become* *more spiritually inclined* as a life-cycle phenomenon [11,12]. However, little research exists on how this inclination relates to their medical care. New evidence suggests that *DT is primarily a spiritual intervention* with implications in the other spheres of a person’s experience.

Figure 1 displays the study’s conceptual framework including the mechanisms to explore. In this individual-level model, we conceptualize physical symptoms and spiritual distress as moderators of the DT effect on spiritual outcomes, measured as dignity impact, existential tasks, and cancer prognosis awareness, and on process measures (patient satisfaction and unmet spiritual needs).

In a study of factors considered important among 340 patients, with advanced illness, most of whom were elderly, some diagnosed with cancer, *being at peace with God was as important as freedom from pain* [2]. This finding is consistent with the broad consensus that attention to patients’ spiritual concerns is 1 of the core dimensions of palliative care and a part of comprehensive geriatrics care [13-15]. Research with terminally ill patients makes clear that, in their view, a good death includes addressing several central spiritual issues and tasks [2,16]. A body of evidence is developing that describes moderate to high levels of spiritual concerns, unmet needs, or struggles among patients in palliative care or with advanced illness [3,17,18]. Existing evidence is limited but points to improved patient illness experience when patients’ spiritual needs are addressed, as well as greater use of hospice rather than ICU care at the end of life [19]. Furthermore, when patients feel that their spiritual needs are unmet, they have lower satisfaction with care and increased emotional distress [20,21].

Delivery of spiritual care services is a variable and the evidence-based studies on chaplaincy interventions are limited. There are few published studies of interventions designed to address the spiritual needs of palliative care patients [22] and *no studies of spiritual care provided by chaplains create a* gaping hole in knowledge of the effects of the interdisciplinary team. Fortunately, professional chaplaincy is currently undergoing an important transformation. Until now, chaplaincy practice has been guided primarily by tradition and expert opinion. However, professional chaplaincy associations recently embraced the importance of evidence-based care, as have practicing chaplains [23]. Presently, the major obstacle that prevents chaplains from adopting evidence-based practice in palliative care is the absence of any research about chaplains’ interventions. Although much of US health care and elsewhere is shifting from a focus on volume-based metrics (eg, number of patients seen or procedures done) to value-based metrics such as increasing patient satisfaction, chaplaincy has been very slow to make this change particularly with regard to proposing and testing appropriate outcomes. In the proposed study, we begin to fill this gap.

Few protocols- or evidence-based chaplaincy interventions have been used to meet the spiritual needs of people facing aging with chronic or serious illness or to test whether meeting those needs would improve outcomes valued by the elderly patient with cancer. *Therefore, rigorous evaluation of a manualized, evidence-based intervention would constitute a landmark step in gero-oncology and palliative care research and in nursing and especially chaplaincy health services research* ***.*** DT is a well-designed, validated, and manualized intervention well-suited for clinical protocolization. For these reasons, we proposed to include both nurse-led and chaplain-led DT with each compared only with usual palliative care and *hypothesize* that each DT group will report higher dignity impact than their respective usual palliative care group, which would mean that either discipline could lead DT when it is implemented clinically in palliative care. Nurses, but not chaplains, have been the DT interventionists in previous feasibility studies; however, we expect both can be trained to competently lead DT in the outpatient palliative care setting with elderly cancer patients. Distinguishing effects between the DT groups is beyond the scope of our application, but our findings will provide effect size estimates for future studies, should such a comparison be warranted by our findings. Both disciplines need to be included in this study to provide an efficient test of the efficacy of DT and time of delivery in clinical care by the disciplines most likely to be available, an important issue for translation of result findings into practice.

### Addressing Intrinsic Limitations to Palliative Care Research by Leveraging Our Network and Using Novel Design

This study is generated from a group of collaborators that came together under the auspices of the Patient-powered Spirituality-and Quality-of-life-focused Advanced-illness Research-network, a group formed in 2013. An open community of 27 organizations (health providers, researchers, patients, and other stakeholders) invited participation in research and health information giving.

Palliative care research is inherently hindered by the patients’ severity of illness. High illness burden and short life spans make participant accrual and retention challenging; the participation burden must be minimal. Using a stepped-wedge design, 1 possible design for an RCT [4], will require a  *smaller number of participants and allow intervention integration into the care process while maintaining power, data quality, and desirable design features including randomization to control and intervention groups.*

In summary, successful achievement of study aims will have important impact on the fields of geriatrics, comprehensive cancer care, and palliative care by showing whether DT can be optimally used in routine care settings to bring better illness experience to patients. It will further advance understanding of how DT works.

### Innovation

This proposed work is innovative in 6 ways. *First*, and most importantly, we primarily conceptualize DT as a spiritual intervention for individuals with serious illness. Other researchers have approached DT as an intervention that broadly improves the dignity of patients nearing the end of life but examined the effects of DT on a variety of outcomes and only psychosocial outcomes showed a significant effect [24,25], In contrast, our *second* innovation is the use of spiritual outcomes as primary and secondary indicators of the DT effect. Our focused conceptualization of DT as a spiritual intervention leads to our choice of unique outcome measures and DT’s focus at the level of the individual patient, though it could be conceptualized at the family level, which is beyond the scope of the proposed study. Other DT researchers used multiple measures of physical and psychological symptoms, as well as a broad measure of personal dignity. In contrast, our measures focus on dignity impact and existential tasks that help patients feel prepared for life completion and to have awareness of their prognosis. Furthermore, our measures of these constructs have been successfully used in previous palliative care or DT-related studies, and our recent reanalysis of RCT data shows that dignity impact differed for the DT group compared with usual care and another patient-centered intervention [5]. Although there is consensus about the central role of spiritual care in palliative care, there are few studies about spiritual care and no studies of spiritual care provided by chaplains. Thus, the *third* innovation of our study is that it employs a rigorous research design to test the efficacy of a manualized nurse-led or chaplain-led intervention in the palliative care context. Studies such as this are essential to clarify what spiritual care contributes to palliative care and to advance an evidence-based approach to chaplaincy care. Consistent with our rigorous test of a spiritual intervention, our *fourth* innovation will be to gather detailed information, in both the usual care and DT samples, about the nurses and chaplains’ assessments and activities for descriptive purposes. The development of evidence-based chaplaincy has been hampered by the lack of evidence-based assessment instruments and standardized descriptions of chaplaincy care. Our work will employ innovative approaches for nurse or chaplain assessment of the patients’ spiritual needs and for nurses’ or chaplains’ description of the care they provide. Prior studies of DT have focused on its effects on key outcomes and patients’ reports of its benefits. They have not proposed or tested hypotheses about the ways that DT improves patient dignity during serious illness like cancer. The *fifth* innovation is the test of 2 new hypotheses about 2 moderators of DT effects, and we will employ rigorous measures to do so. The *sixth* innovation is that we will employ a stepped-wedge design, which has been used in other fields, but is new to clinical trials in the palliative care context and addresses the challenges of recruitment in this clinical setting.

In summary, by focusing on DT as a spiritual intervention, using a new conceptual model and measures consistent with the model to examine its effect, examining several pathways that shape its effect, and using a stepped-wedge design, our study will contribute important novelties to palliative care research. In addition, by testing the effects of nurse-led or chaplain-led DT, we will advance an evidence-based approach to spiritual care in the gero-oncology palliative care context, which is highly responsive to the National Institute of Aging’s and the National Cancer Institute’s request for palliative care applications (PA-13-354/NOT-CA-14-016) to study elderly cancer patients.

### Approach

#### Study Team

Our study has a high probability of success because we will apply an interdisciplinary approach with highly productive investigators possessing expertise in palliative medicine, chaplaincy health services research, and nursing research focused on palliative care topics. The multiple principal investigators, MPIs (LE, GF, and DW), and coinvestigators, Co-I (MC, GH, and YY) have worked together within the HealthCare Chaplaincy Network and on palliative care research and publications for many years [26].

#### Preliminary Studies

Harvey Chochinov, MD PhD, originator of DT and lead investigator on many of its main studies, is a highly involved member of our team and provided data analysis of the dignity impact measure [5]. In addition, we and other research groups have established its feasibility among seriously ill patients, including those receiving hospice or palliative care. As we recently reviewed [26], 12 studies of DT established it as an important intervention: 8 uncontrolled feasibility studies (3 with 29 to 100 patients) and 4 efficacy RCTs (3 with less than 65 patients; Table 1). Taken as a group, 2 findings stand out. *First*, DT does not have consistent effects on distressing physical or emotional symptoms experienced by patients with life-threatening illness. As Hall et al [24] noted, 1 possible explanation for these inconsistent findings is that DT does not directly affect physical symptoms and thus it is a mistake for DT research to focus on physical symptoms or related outcomes as primary success indicators. *Second*, patients who receive DT provide uniformly high ratings of satisfaction and benefits for themselves and *report significantly higher dignity impact ratings* than patients who receive usual care or another patient-centered intervention [5]. It is important that *all* the existing studies consistently show feasibility of DT, and 1 study shows promising evidence that dignity impact is an appropriate outcome [5]. These findings are important as dignity impact is important to patients, especially the elderly with life-threatening illness, such as cancer. In addition, DT increased patients’ *sense of meaning and purpose and their will to live* [27], which is important for patients continuing cancer treatments and participating in cancer research (both are emphases of NOT-CA-14-016).

In summary, we propose conceptualization of DT as a spiritual intervention that assists with the existential tasks faced by elderly patients as they face a serious illness like cancer. This conceptualization leads us to 2 additional decisions. First, nurses and chaplains are the appropriate members of the palliative care team to offer DT. As prior studies had the DT delivered by palliative care nurses or other health professionals, showing that the efficacy of DT is significantly better than usual care, whether the DT is delivered by chaplains or nurses, would be a scientific and clinical advance for translation of DT into geriatric and oncology practice. Second, although they have not been used in prior DT studies, measures of the existential tasks associated with life-threatening illness, as well as dignity impact, are appropriate DT outcome measures.

Although there is limited research about the benefits of chaplains’ care [33] and no research about the benefits of chaplains’ care in palliative care, for decades chaplains have understood their important role in helping patients with existential tasks associated with serious illness [34]. As part of a project funded by the Templeton Foundation and led by members of our team (Emanuel, Handzo), 6 studies of chaplaincy care have recently been completed and published [35,36]; others are being prepared for publication. None of these studies tested the effects of chaplain-led DT on patients’ existential preparation for death, but one documents that chaplains are willing and able to use a manualized intervention. Thus, our proposed work will fill 2 unique gaps in DT research: the effects of DT on important existential tasks associated with serious illness; and chaplains’ contributions to improving the care of those patients.

Under MPI Dr LE’S mentorship, Vergo conducted a feasibility study of DT early in the illness course of outpatients with stage IV colorectal cancer receiving second line chemotherapy (Table 1, study 4) [6]. In addition to outcomes on distress, symptoms, and quality of life; relevant outcomes also included peaceful awareness and treatment preferences. Of patients approached to participate, 88% did so. The findings suggest not only feasibility of DT during cancer care, but some improved physical and emotional symptoms, increased understanding of the terminal nature of their disease, less aggressive end-of-life goals of care, and increased death acceptance over time (11% at baseline; 57% at 1 month post-DT). These results are suggestive of improved spiritual as well psychological, social, and physical states and give our team recent experience with DT for patients with cancer undergoing active cancer treatments.

Working with Chochinov, we recently reanalyzed data from the study of 441 Canadian, Australian, and US hospice or palliative care patients [1] using the 7-item dignity impact scale we propose as our primary outcome. We found strong internal consistency (α=.85) and that the DT group mean score of 21.4 (SD 5.0) was significantly higher than the usual care group mean 17.7 (SD 5.5, *P*<.001) and a patient-centered intervention group mean of 17.9 (SD 4.9, *P*<.001) [5]. These preliminary findings provide strong support for our proposed 3-arm efficacy trial because they indicate that the DT affects the dignity impact, not an artifact of the attention provided during individualized therapy because the patient-centered intervention group did not show an effect better than usual care. Our prior studies provide solid evidence that the proposed 3-arm study will be successful: access to an ethnically diverse population of patients with cancer and receiving outpatient palliative care, prior success recruiting and retaining in a DT trial of colorectal cancer during cancer care, a well-tested intervention training program and manualized intervention, fidelity measures, and outcome measures that are robust in detecting the effect of DT. We are now poised to learn if nurse-led or chaplain-led DT is more effective than usual care to improve dignity impact as the primary outcome. Our secondary outcomes were sensitive to other psychosocial interventions [37-39], but have not been used in previous DT RCT studies, which is why we do not propose them as primary outcomes. We also have used the measures for moderator effects. Therefore, our preliminary work is strong and warrants the proposed RCT.

**Table 1:** Prior trials of dignity therapy.

Study and sample		Design, measures, and interventionists	Findings
**Feasibility studies with N≥15**			
	100 Canadian & Australia terminally ill[28]	Design: Pre-post trial of dignity therapy (DT); Measures: Single item screening measures for 8 factors (depression, anxiety, suffering, suicide, sense of well-being; QoL^a^, ESAS^b^; DTPFQ^c^); Intervention: psychiatrist, psychologist, and palliative care nurses	Significant improvement in suffering and depressed mood. High proportions gave positive evaluation to benefits of DT (eg, 91% feel satisfied or highly satisfied with DT, 86% report DT was helpful or very helpful)
	80 Danish cancer patients in hospice or palliative care [29]	Design: Pre-post trial of DT; follow up after (T1) & 1 mo after (T2); Measures: SISC^d^; PDI^e^: EORTC QLQ-C15-PA^f^; HADS^g^; PPSv2^h^; DT PFQ; Intervention: psychologists	No change on any measure at T1 or T2 except QoL decreased baseline to T1. At T1 and T2, positive responses on DTPFQ
	29 Australian patients with MND^i^ [30,31]	Design: Pre-post trial of DT; Measures: Hope; FACIT-Sp^j^; PDI; DT PFQ; ALS^k^ measures; Intervention: psychologist	Feasibility and acceptability established. High satisfaction (93%) and helpfulness (89%) for DT. Not significant: hope, spirituality, and dignity.
	15 US stage IV colon cancer patients, active cancer treatment [6]	Design: Pre-post trial of DT; follow up after DT (T1) & 1 mo after (T2); Measures: ESAS; distress; QoL; peaceful awareness; advanced care planning; DTPFQ (selected items) Intervention: palliative care oncologist	Feasibility and acceptability established. High satisfaction (100%) and helpfulness (88%) for DT. No significant changes in other study measures.
**Efficacy studies**			
	441 Canadian, Australian, & US hospice or palliative care[1]	Design 3 arm RCT: DT vs client-centered vs standard care; Measures: SISC; ESAS; PDI; QoL- 2 items; HADS; FACIT-Sp; DTPFQ; Intervention: psychiatrist, psychologist, and palliative care nurses	No significant differences on any outcomes. Reanalysis of dignity impact items: DT group has significantly higher scores than standard care (*P*<.001) or client-centered care (*P*<.001).
	45 UK advanced cancer[24]	Design RCT: Tx = DT plus usual care; Control=usual care (Phase II trial for acceptability and estimates of effect sizes); Measures: Primary: PDI; Secondary: Hope; HADS; EQ-5D^l^; palliative-related outcomes (Hearn); DTPFQ; Intervention: oncologist	No differences on PDI. No differences on any secondary outcomes, except higher hope in group at week 1 (*P*=.02). Patients in the DT group had higher scores on DTPFQ, some significant.
	64 UK patients in older care homes[32]	Design RCT (Phase II trial for potential efficacy, feasibility): Tx = DT plus usual care; Control = usual care; Measures: Primary: PDI; Secondary: GDS^m^, HHI^n^, EQ-5D, Acceptability: DTPFQ ; Intervention: palliative care nurse	No differences on efficacy outcomes; reduced dignity-related distress on DTPFQ across both groups (*P*=.03). DT group significantly more likely to feel DT had made life more meaningful at follow up 1 (*P*=.04).
	60 Portuguese terminally ill[25]	Design RCT: Tx = DT+usual care; Control = usual care; Measures: HADS; Intervention: palliative care physician	DT associated with lower depression and anxiety (day 4 and 15, not day 30; all *P*<.05)

^a^QoL: quality of life.

^b^ESAS: Edmonton System Assessment Scale.

^c^DTPFQ=DT patient feedback questionnaire.

^d^SISC: Structured Interview for Symptoms and Concerns.

^e^PDI: Personal Dignity Inventory.

^f^EORTC QLQ-C15-PA: European Organization for Research in Cancer Quality of Life Questionnaire-C15-Palliative.

^g^HADS: Hospital Anxiety Depression Scale.

^h^PPSv2: Palliative Performance Scale.

^i^MND: motor neurone disease.

^j^EQ-5D: EuroQol group’s five dimensions.

^k^FACIT-Sp: Functional Assessment of Chronic Illness Therapy-Spiritual Well-being.

^l^ALS: amyotrophic lateral sclerosis.

^m^GDS: Geriatric Depression Scale.

^n^HHI: Herth Hope Index.

## Methods

### Design

**Figure 2:**
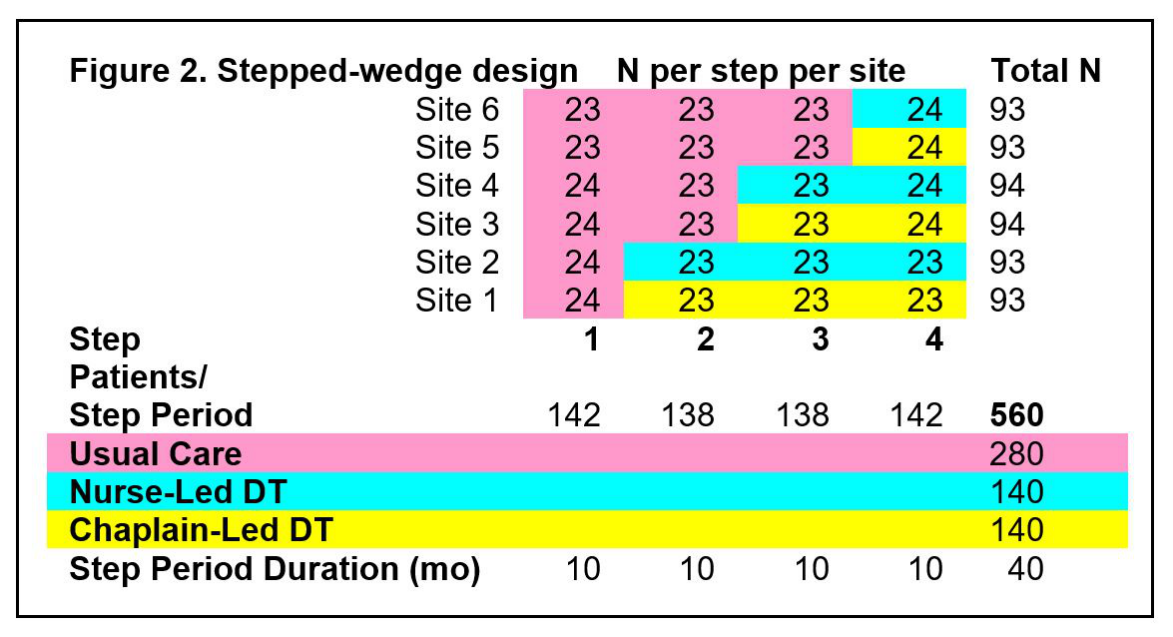
Stepped Wedge Design.

We propose a 6-site, pre- and posttest, randomized, controlled 4-step, stepped-wedge design to compare the effects of usual outpatient palliative care and usual outpatient palliative care along with nurse-led or chaplain-led DT on patient outcomes and palliative care processes. We will assign the 6 sites to usual care during the first-step period (10 months), and randomly assign 2 sites per step to begin and continue DT during each of the next 3 steps (10 months each). Figure 2 shows the stepped-wedge study design with projected numbers of completed patients needed per site, step period, and group (usual care, DT led by either a nurse or a chaplain). Dr. Yao, a highly qualified statistician, will conduct the randomization of site from steps 2 to 4. Each step will be 10 months long, with DT training during a 1-week period between steps. For each site and step, a quota of 50% of the participants will report low or high distress on the Personal Dignity Inventory to assure that we recruit a sample with a range of problems threatening their dignity. Each patient will participate for 4 to 6 weeks. During the 10 months at each step, we expect 23 to 24 patients to participate at each site (93 to 94 total patients per site).

### Setting

For this efficacy study, the settings will include Northwestern University Hospital, Rush University Medical Center, MD Anderson Cancer Center, Emory University, University of California San Francisco, and University of Florida Health. The clinical resources of each of these settings are strong. Our team members have extensive experience recruiting and retaining elderly patients with cancer for studies, including palliative care and chaplaincy research.

### Sample

We will recruit the study sample from the populations receiving care at settings across the United States. For the patients who meet the eligibility criteria, we anticipate a 50% to 60% enrollment rate and a 20% to 30% attrition rate. We based these estimated numbers on accruals, withdrawals, and deaths in previous palliative care studies conducted at study settings and those in prior studies of DT. During the 40 months devoted to data collection, we anticipated a total available population of 3126 from the outpatient palliative care services of the 6 sites. From this sample, 560 patients will complete sufficient data for the planned analysis, 140 per each of 2 DT groups and 280 total control group. Therefore, we expect each of the 6 sites to complete at least 2 to 3 patients per month and a final total of 93 to 94 patients per site with data for analysis. On the basis of ethnic and racial distribution of patients served at the 6 sites, we expect the final sample to be 50% female, about 3% Asian American, 21% African American, 73% Caucasian, and 3% unknown race and 8% Hispanic ethnicity, representing substantial cultural diversity for this study of DT.

### Eligibility Criteria

*Inclusion* criteria for study participation require that the patient (1) has a cancer diagnosis (receiving cancer therapy or cancer control care to be responsive to NOT-CA-14-016), (2) is receiving outpatient palliative care, (3) is aged 55 years or older (responsive to PAR-13-354), (4) is able to speak and read English, and (5) is physically able to complete the study (Palliative Performance Scale [PPS]>50 [40-45], suggesting a mean in life expectancy of greater than 53 days at the time of enrollment) [45]. Patients will be *excluded* if they: (1) are legally blind, (2) are cognitively unable to complete study measures (Mini Mental Status Exam [MMSE] <24), (3) have history of psychosis (medical record review), (4) have a Patient Dignity Inventory (PDI) or Religious and Spiritual Struggles Scale score that indicates their distress level falls outside the remaining quota for a given step; quota is 50% of sample, site, and step with low distress (≤ 2 problems rated >2) and 50% with high distress (≥ 3 problems rated >2 or 1 rated>3), or (5) are enrolled in another intervention study that is focused on concepts similar to the proposed study.

### Retention Strategies

Retention strategies are vital to engaging participants as active research partners. Researchers in this study will always use respectful, empathic communication and schedule data collection at convenient times for participants. We will collect multiple contact information (email, cell phone, home, and extended family phone) to maximize our ability to contact them for final appointments. We also will offer a total of US $50 per patient to cover time and travel expenses to complete the study measures.

### Sample Power

*Aim 1* focuses on whether DT has an effect on patient outcomes and palliative spiritual care processes. For the 4-step, stepped-wedge design and the sample size of 23 to 24 patients per site per step (93 to 94 total), we determined from the minimum intervention effect size that the proposed study has at least 80% power to detect given a 2-sided significance level of .5. This minimum detectable effect size depends on disparity between sites as indexed by the intraclass correlation (ICC). With no site disparity (ICC=0), we can detect an effect size of 0.5 or above for either the nurse-led or chaplain-led DT intervention; with a more severe disparity (ICC=.4), we can detect an effect size of 0.6 or above. The *primary outcome* for this study is the 7-item Dignity Impact scale. Using Dignity Impact scale data from the preliminary study [1], the effect size of DT (relative to usual care) was 0.7 [5]. Therefore, we expect this study to have sufficient power to detect the intervention effect on the primary outcome. We do not have preliminary DT data on the secondary outcome measures; however, based on other psychosocial interventions with the measures [37-39,46], our power analysis indicates that as long as the intervention effect on these outcomes is of medium size (0.5 as defined by Cohen) or larger, they can be detected in the proposed study. Our study aims to focus on differences between the control group and each DT group, but do not focus on differences between the provider groups because, with the proposed sample, the *effect size difference between the nurse-led and chaplain-led DT groups needs to be large (0.7 when ICC=0 and 0.9 when ICC=.4)* to be detectable (with 80% power). As prior research shows an effect size of 0.7 between control and DT groups, it is highly unlikely that there will be a significant difference between the provider groups with the proposed sample. *Aim 2* seeks to explore whether or not patient distress (physical or spiritual) moderates the effect of the intervention. The intervention effect size of patients with high distress will be compared with that of patients with low distress level. The study has power (80%) to detect an effect size difference of 1.0 if there is no site disparity (ICC=0) and 1.3 if there is severe site disparity (ICC=.4). Assuming an average effect size of 0.8 and group balance, we can detect a moderation effect if the intervention effect size for the less receptive group is 0.15 or lower.

### Procedures

**Figure 3:**
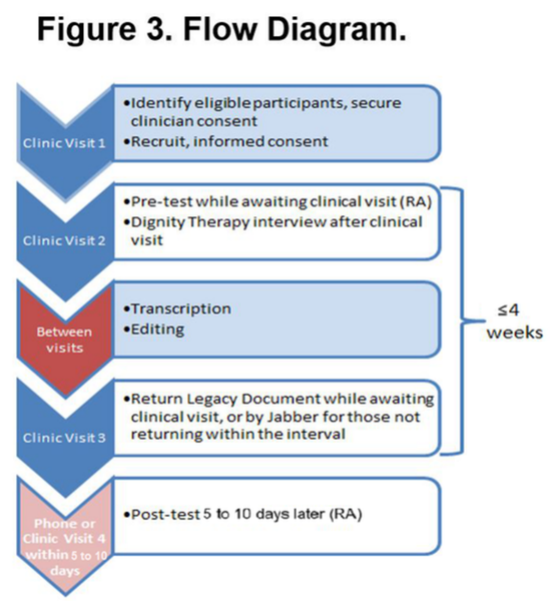
DT Study Flow.

Figure 3 shows the general flow of patients through the outpatient palliative care clinics and study procedures for this study of nurse-led or chaplain-led DT over 4 to 6 weeks. Before participant recruitment, the research assistants (RAs) will be trained in all study procedures. The investigators will introduce the study to the palliative care team members. Members of the palliative care team will refer patients to the RA on the day the patient is seen in the palliative care clinic.

The RA will recruit and consent the patient for screening. For those eligible, the RA will provide informed consent and schedule time before the next clinic visit for the patient to complete the pretest self-report measures using a tablet with smart pen. Then, the RA will inform the nurse and chaplain that the patient is available for care (usual care or DT, depending on the study step and site). The nurse or chaplain will provide the designated care on the same day as the pretest data collection. When the nurse or chaplain sessions are completed, the nurse or chaplain will inform the RA that he or she has completed all nurse or chaplain sessions. Either in-person if the patient has a clinic visit or by telephone contact, the RA will schedule the follow-up appointment and complete the posttest measures within 5 to 10 days. Upon completion of posttest measures, the RA will provide a US $50 cash per card to the patient for time and travel expenses to complete study measures.

**Examples of the Dignity Therapy Question Protocol.**

1. Tell me about your life history; particularly the parts that you either remember most or think are the most important? When did you feel most alive?

2. Are there specific things that you would want your family to know about you, and are there particular things you would want them to remember?

3. What are the most important roles you have played in life (family roles, vocational roles, community service roles, etc)? Why were they so important to you, and what do you think you accomplished in those roles?

4. What are your most important accomplishments, and what do you feel most proud of?

5. What are your hopes and dreams for your loved ones?

6. What have you learned about life that you would want to pass along to others?

7. What advice or words of guidance would you wish to pass along to your [son, daughter, husband, wife, parents, other(s)]?

8. Are there particular things that you feel still need to be said to your loved ones, or things that you would want to take the time to say once again?

9. Are there words or perhaps even instructions you would like to offer your family, in order to provide help to prepare them for the future?

10. In creating this permanent record, are their other things that you would like included?

### Interventions

In this trial, we will compare the efficacy of 2 interventions, both provided either by palliative care nurses or chaplains. The nurses will be the registered nurses who are trained and working with the palliative care team. Chaplains will be BCC. To become a BCC, a person must have completed a graduate-level theological education as well as 1600 hours of supervised practice in an accredited program of clinical pastoral education. BCCs must be in good standing with, and endorsed by, their faith group. In their application for Board Certification and interview, BCC candidates must demonstrate competence in 29 areas. BCC chaplains adhere to a code of ethics that includes “respect for the cultural and religious values of those they serve and refrain from imposing their own values and beliefs on those served.” BCCs must complete 50 hours of continuing education annually and a peer review every 5 years [47].

### Usual Care

Palliative care nurses usually see patients each clinic visit to assess vital signs, function, symptoms, and to provide patient and family education. They document findings and interventions in the electronic health record (EHR). Although, usual care for palliative care chaplaincy in the outpatient setting varies by site, chaplaincy care for usual care patients in this study will follow the usual practice.

### Dignity Therapy

The DT intervention is detailed in Chochinov’s manualized guide; he serves as a coinvestigator. He advised the development of this application since its inception and gave us permission to use the manualized intervention as it appears here in abbreviated form. The basic questions of the DT interview appear in Textbox 1. The nurse-led or chaplain-led DT intervention involves 3 sessions, each of which follows a set process (Table 2). Nurses are familiar with use of manuals. Chaplain use of manuals is not common, but Dr. Karen Steinhauser (personal communication, February 2014) confirmed that trained chaplains used a manualized intervention guide with high fidelity. Table 3 lists exemplars of the dignity repertoire (perspectives and practices) facilitated by the interview and document preparation process. The standardize approach to the delivery of the intervention facilitates a personal process of reflection and recognition that allows the patient to make meaning of their experience.

### Dignity Therapy Training

Dr. Chochinov, who developed DT, will provide standardized training for the study nurses and chaplains at one of the study sites on 3 separate occasions; each training session will be 2 days in duration. Nurses and chaplains at all sites will be prepared to participate in the DT training during the 3 training dates that will be set early in year 1 but will first occur at the end of year 1. Only those nurses or chaplains at the randomized sites randomized to begin DT at the next step will be trained at any one date, and they will be notified before the training date. By the 4th step, all nurses and chaplains will be trained, assuring competency of all interventionists to deliver DT with fidelity. If there are new nurses or chaplains at a site randomized to the DT step, they will be allowed to attend remaining training sessions. In addition, 2 weeks before the end of each step, the statistician will divulge the 2 sites randomized to the next step. Before training, the MPI will inform the team members about the randomization. To reduce potential influence of the nurse or chaplain anticipating the training and thereby altering usual care practice, we will suspend recruitment, pretest data collection, and intervention delivery during the training periods; posttest data collection will continue.

### Intervention Fidelity

The second RA, who is not involved with data collection, will complete the DT Adherence Form as a measure of intervention fidelity for all DT patients. Members of our team used this tool in previous studies with excellent results. The RA will read each original DT transcript and use this measure to document the nurse’s or chaplain’s adherence to the DT protocol. Scores range from 0 to 10, with 10 representing complete adherence. We will monitor the adherence scores to determine if a nurse or chaplain requires additional training by Rev. Handzo (those with scores <8).

**Table 2:** Dignity therapy intervention ingredients.

Session	Timing	Purpose	Key Ingredients (Features)	Process Considerations and Issues
*First:* nurse-led or chaplain-led Diginity Therapy (DT) contact (information session)	Visit 1	To establish relationship with patient; to explain DT history and procedures	DT is based on piloted studies. Sessions are tape-recorded, transcribed, edited, and returned to the patient for feedback. Process is iterative. Purpose is a legacy generating document for family or friends. DT can be free form, guided, or both. Guide questions may be provided before second meeting upon request. Recording session is scheduled.	Rapport must be established with patient. Patient must understand the process. Nurse or chaplain should be knowledgeable of process. Nurse or chaplain should have guide questions available for patient.
Second nurse-led or chaplain-led dt contact (recording session)	Visit 2: + 2 weeks	To provide DT; to record DT session	Tape-recorded DT session begins with either patient directed content or guide questions. Session takes about 60 minutes and is highly flexible, accommodating the patient’s desired discussion content. Nurse or chaplain takes an active role, forming a therapeutic alliance while delivering and organizing the structured intervention. Legacy document session is scheduled.	Nurse or chaplain must maintain respect, empathy, support, and dignity.
Intermission (No contact)	Nonvisit: 2-4 weeks	To transcribe the session; to edit the manuscript; to revise the manuscript; to produce a legacy document	Nurse or chaplain must guide without providing judgment statements. Tape recorder should be tested before session. Recording session is transcribed by a professional transcriptionist. Three copies are kept: a) unedited complete transcript, b) ‘tracked’ version of the edited transcript, and c) final edited version. Single editor initially edits the manuscript: cleaning up the colloquialisms and nonstarter stories, adjusting the chronology, and removing stories that may be hurtful or harmful. Nurse or chaplain reviews the document, making changes with the editor. Final edited manuscript will end with a summary phrase driven by the patient’s story.	Nurse or chaplain read transcription copy for accuracy before editing. Editor must remain unbiased while editing, making sure the themes come through without changing the content. Editor must choose an ending to summarize the patient’s story without biasing content. Timeliness is important.
Third nurse-led or chaplain-led DT contact (Legacy document session)	Visit 3: +4 weeks	To deliver edited legacy document; to receive feedback from patient	Nurse or chaplain delivers final edited legacy document to the patient. Nurse or chaplain reads it to the patient or the patient will read it alone. Patient may request editorial changes which will be completed within 24 hours. If revisions are necessary, nurse or chaplain makes arrangements for the final delivery of the legacy document within 24 hours. Patient makes arrangements to deliver the legacy document to loved ones.	Editing may not satisfy the patient. Theme may not be approved by patient. Patient may not be able to provide feedback
Final editing (if necessary)	Nonvisit: 24 hours post Visit 3	To make final revisions to legacy document	Nurse or chaplain makes final revisions based on patient feedback and delivers the final legacy document to the patient.	Final revisions are not approved by patient (process closure)

**Table 3:** Dignity therapy: exemplars of repertoire (perspectives and practices) facilitated by interview and document preparation process.

Dignity conserving Repertoire	Ways of looking at one’s situation, or personal actions that can bolster or reinforce a sense of dignity
Dignity conserving perspectives	Internally held qualities, often based on long standing personal characteristics, attributes, or world view
Continuity of self	A sense that the essence of who one is continues to remain intact, in spite of one’s advancing illness
Role preservation	Ability to continue to function in usual roles to maintain a sense congruence with prior views of self
Generativity and legacy	The solace and comfort in knowing that something lasting will transcend their death
Maintenance of pride	The ability to maintain a positive sense of self regard or respect
Hopefulness	An ability to see life as enduring, or having sustained meaning or purpose
Autonomy and control	A sense of control over one’s life circumstances
Acceptance	The internal process of resigning one’s self to changing life circumstances
Resilience or fighting Spirit	Mental determination to overcome illness-related concerns and optimize quality of life
Dignity conserving practices	Variety of personal approaches or techniques that patients use to bolster or maintain their sense of dignity
Living in the moment	Focusing on immediate issues in the service of not worrying about the future
Maintaining normalcy	Continuous or routine behaviors, which help individuals manage day-to-day challenges
Seeking spiritual comfort	Turning toward or finding solace in one’s religious or spiritual belief system

### Measures

All our measures have been used among palliative care populations, summary of timing of these measures is shown in Table 4. To collect these data, we will create a REDCap study site using the UF REDCap system, which the UF CTI supports and makes available for NIH funded studies without cost. All data collection sites will use this secure data collection site. Patients will use a tablet with a smart pen to enter the data into the REDCap system. Wilkie et al are successfully using this cost-effective approach to data collection for another palliative care study [48] focused on African American caregivers and the data manager is well versed with REDCap and current mobile devices. Wilkie et al is also successfully using mobile tablet or pen devices in a study of hospice patients in their homes and replicating prior findings of the clear feasibility of elderly patients with cancer near the end of their lives using the tablet technology, especially with use of a smart pen device [49]. We expect that patients will take 30 to 45 minutes to complete the measures at pretest and 15 to 20 minutes at posttest.

### Patient Outcomes

The primary study outcome is dignity impact with all study measures. The secondary outcomes are existential tasks and cancer prognosis awareness.

### Dignity Impact

Our primary outcome measure is a 7-item Dignity Impact Scale [5]. We took the items from the DT Patient Feedback Questionnaire that has been used in a number of studies of DT [50]. The 7 items addressed the concept of DT and showed significant differences between those who received DT and those who did not in an RCT [1]. The items have been used in many DT studies with evidence of their validity in the target population. We modified the wording of the items to fit our pretest and posttest study design. A sample item is “The care I received during the past month has increased my sense of dignity.” The items are scored on a 5-point scale from “strongly disagree” (1) to “strongly agree” (5). The Cronbach α from a preliminary study was .85 [5]. Scores ranged from 7 to 35 in the DT group and 7 to 29 in the usual care group and showed sensitivity to DT effects at posttest with an effect size of 0.7 [5]; it was not measured at pretest, but we will do so to increase power and potential for inferences.

### Existential Tasks

The existential tasks of importance in the proposed study are preparation for death and life completion. Our measures of preparation and completion are taken from the QUAL-E, a measure designed to evaluate the quality of life at the end of life and to assess the effectiveness of interventions targeted to improve the quality of life at the end of life [46]. The 4-item Preparation subscale assesses an individual’s sense of integrity and concerns about being a burden to significant others. A sample item is “I have regrets about the way I have lived my life.” The 7-item Completion subscale assesses an individual’s sense of meaning and peace, as well as any unfinished interpersonal business. A sample item is “I have been able to share important things with my family.” The items are rated on a 5-point scale from “not at all true for me” (1) to “completely true for me” (5). The items in the Preparation subscale are reverse scored. The Preparation and Completion scales have demonstrated good reliability and validity [46]. Validity of both measures was shown by correlations in expected directions with other measures of quality of life (FACT-G; Missoula-Vitas QOL) and spiritual well-being (FACIT-Sp). In a diverse sample of 248 patients near the end of life, the Cronbach alphas were .68 and .80 for Preparation and Completion, respectively. The 1-week ICCs for participants with no changes in health status were .73 for Preparation and .72 for Completion.

**Table 4:** Measures, time points, and person who completes.

Measure (concept-aim); [number of items]	Pretest	Posttest
Dignity impact (Aim 1)	X^a^	X
Quality of Life at End of Life (QUALE-E) Existential tasks-aim 1)	X	X
Cancer prognosis awareness (Aim 1)	X	X
Treatment preferences (Aim 1)	X	X
Patient satisfaction with chaplain and nurse care (Aim 1)	X	X
Edmonton symptom assessment scale (physical symptoms-aim 2)	X	—^b^
Religious and spiritual struggles scale (spiritual distress-aim 2)	X	X
Demographic and patient characteristics	X	—

^a^X: data collected.

^b^—: data not collected.

### Cancer Prognosis Awareness

Two measures will provide data for peaceful awareness and treatment preference. Our approach to measuring terminal illness awareness (peaceful awareness) is taken from a study of 280 patients with advanced cancer who participated in the Coping with Cancer Study [51]. In that study, 17.5% of the sample reported being both peaceful and aware of their prognosis. Peacefully aware patients had lower rates of psychological distress and higher rates of advance care planning than those who were not peacefully aware. Peaceful awareness is also associated with modifiable aspects of medical care (eg, discussions about terminal treatment preferences).

*Treatment preferences* will be measured with a standardized and validated Hypothetical Advanced Care Planning Scenario that assesses scenario-based goals of care and treatment preferences. This approach, based on work by MPI LE [52], was used successfully in the study of DT in patients with advanced colorectal cancer [6]. Patients will be considered as making “life prolonging” treatment choices if they selected “I want” or “I want treatment tried. If no clear improvement, stop (only for mechanical ventilation)” for either CPR or mechanical breathing. Patients will be considered as making “non-life prolonging” treatment choices if they selected “I do not want” to both CPR and mechanical breathing. Patients selecting “I am undecided” to either CPR or mechanical ventilation and not considered “life-prolonging” in their treatment choices will be categorized as “undecided.”

### Palliative Care Spiritual Service Processes

We will measure 2 indicators of palliative care spiritual service processes. They are patient satisfaction with spiritual care and unmet spiritual needs.

### Patient Satisfaction

The items in our 7-item measure of Patient Satisfaction with Nurse or Chaplain Care are taken from the work of 2 leaders in chaplaincy research. VandeCreek [53] developed the 23 item Patient Satisfaction Instrument-Chaplaincy (PSI-C) that he administered to a sample of 1440 patients who had been treated in one of 14 different US hospitals. In their study of patient satisfaction, Flannelly et al [54] used 7 items from VandeCreek’s PSI-C as well as 7 new items they developed. In their study, with 250 patients at 1 hospital, the validity of their items was supported by positive correlations with other items that assessed patients’ perception that their spiritual and emotional needs had been met. Following the work of Flannelly et al [54], we will assess 2 aspects of satisfaction; satisfaction with the process of nurse or chaplain care and satisfaction with the impact of the chaplains’ care.

### Unmet Spiritual Needs

As clinicians embraced an evidence-based approach to spiritual care, 1 of the challenges they faced is developing instruments that can be used for spiritual assessment [55].

#### Covariates

The covariates for this trial include physical symptoms, measured by the revised version of the Edmonton Symptom Assessment Scale (ESAS-r), and spiritual distress, measured by the Religious and Spiritual Struggles Scale as described in the previous section. The ESAS-r is a well-validated and widely used instrument to assess common symptoms (eg, pain, fatigue, loss of appetite, and shortness of breath) in palliative care patients [56,57]. Each symptom is rated on a scale ranging from 0 to 10, where 0=no symptom and 10=worst possible symptom. The assessment is usually completed by the patient independently. However, the RA will help the patient complete the assessment if he or she is unable to rate all the symptoms.

#### Descriptive Variables: Characteristics of Patients and Nurse or Chaplain Spiritual Activities

Via self-report or medical record review, we will collect the patient demographic variables: age, sex, ethnicity, race, education level, marital status, medical diagnoses, health problems, cancer diagnosis date, type, and stage (at diagnosis and at study enrollment), and ongoing cancer treatments (eg, radiation, surgery, chemo, and immunotherapy). We will collect information about the participant’s religious and spiritual involvement (eg, religious affiliation and frequency of prayer) using standard items [58].

#### Screening for Eligibility and Exclusion Criteria

The PPS, a modified version of the Karnofsky Performance Scale [59], measures 5 functional domains: ambulation, activity and evidence of disease, self-care, intake, and level of consciousness. Its scores range in 10% increments from 0% to 100%, with a score of 0% indicating death, 10% indicating a totally bed-bound patient who is unable to do any activity and needs total assistance, and 100% indicating the patient is able to carry on normal activity and to work without any special care. The findings from studies conducted around the world and 4 regions of the United States [45] support very strong validity with the KPS [43] and adequate interrater reliability [45]. The PPS has predictive validity for average survival of 53 days at the eligibility score for the proposed study (>50) [40-45]; study participation is 28 to 42 days maximum.

We will use the MMSE for cognitive status screening. The MMSE [60] is a valid and reliable quantitative measure of the patient’s cognitive performance and capacity. Rated by the trained RA, it measures on a scale of 0 to 30 a variety of cortical cognitive functions. An MMSE score of 26 to 30 represents a normal range, 20 to 25 represents mild cognitive impairment, and scores below 20 represent moderate to severe impairment. Patients with MMSE scores of <24 [61] will be excluded.

We will use the PDI to assess the level of dignity-related distress in the participants to ensure that a reasonable number of patients experiencing some dignity-related distress are recruited at each step. The PDI contains 25 items that assess a broad spectrum of end-of-life distress including physical, psychological, existential, and spiritual sources of distress [62]. The construct and face validity, test-retest reliability, and factor structure of the PDI have been established [62]. Patients rate each item on a 1 to 5 scale (1=not a problem, 2=somewhat of a problem, 3=a problem, 4=a big problem, and 5=an overwhelming problem). Using a score >3 to indicate a problem for that item, 253 patients receiving palliative care reported an average of 5.7 problems (SD 5.5, range 0-24 problems) [63].

### Statistical Analysis

Data management and preliminary data analysis procedures will be supervised by MPI DW and conducted by Dr. YY, the Co-I, and statistician, using statistical software R version 3.5.2 (R Foundation for Statistical Computing). Data will be stored in a REDCap database and will be exported to R. In the case of missing data, multiple imputations will be used to generate multiple complete datasets on which statistical inference will be performed and then aggregated. Missing at random assumption will be assessed and if necessary sensitivity analysis will be performed using pattern mixture methods. We will consider a *P* value less than .05 as statistically significant.

Descriptive statistics (ranges, frequencies, means, and SDs) and graphic summary (box plots, histograms, bivariate scatterplots, etc) will be first generated for both patient covariates and outcome measures. We will check patient characteristics to see if there is notable imbalance between the 3 arms, as well as whether there are significant variations between sites or over time. Descriptive statistics of patient outcomes and process data will reveal patients’ spiritual state, identify potential areas for improvement in nurse and chaplain service, and provide information on nurses’ and chaplains’ workload both in usual care and when dignity therapy is added and on family related variables (eg, legacy document disposition).

#### Aim 1

Linear mixed effects models will be used to compare the effects of usual palliative care with usual palliative care with nurse-led and chaplain-led DT groups on (1) *patient outcomes* and (2) *processes* of delivering palliative spiritual care services. Random effects terms will be used to model the variations between sites. A main challenge of data analysis of a stepped-wedge design is the modeling of potential time trends. We plan to treat time as a continuous variable and utilize smoothing splines to model potential time trends. Smoothing splines is a nonparametric method that allows flexibility to model different time trends without overfitting by enforcing a smoothness constraint. Likelihood ratio tests will be used to determine the statistical significance of the intervention effect on various outcome measures. We hypothesize that, controlling for pretest scores, patients in both nurse-led and chaplain-led DT groups will have higher dignity impact scores, higher preparation for death and life completion scores, better peaceful awareness, and treatment preferences more consistent with their cancer prognosis than the usual care groups. We also expect the patients in the DT groups to be more satisfied with the palliative care and have fewer unmet spiritual needs than the usual care groups. We do not expect significant difference between nurse-led and chaplain-led DT interventions. Note that, this does not necessarily mean that the 2 interventions are equally effective. We do not seek to test the equivalence of the 2 intervention arms. At this point, it is premature to speculate on their relative efficacy. We expect most patients in this study to have metastatic cancer. If a sizable portion of our sample has nonmetastatic cancer, we will explore if they respond to DT differently than those with the metastatic disease.

#### Aim 2

The effect of physical symptoms and spiritual distress on the dignity impact and existential tasks will be modeled nonparametrically using smoothing splines. Our models will include both main effect for physical symptoms and spiritual distress and their interactions with the intervention. This analysis will provide insight on the type of patients most in need of and most likely to benefit from the DT intervention. Likelihood ratio tests will be used to determine the significance of the interaction. We hypothesize that patients’ levels of spiritual distress and physical symptom scores will moderate the intervention effect.

#### Timeline

Table 5 presents the basic timeline for general activities to achieve study aims. We will prepare study materials, train, and calibrate staff upon award. DT training will commence at the end of month 15. The step-up to step2 occurs in month 16 (12 months per step). Data will be collected from months 4 to 54, processed starting month 4, analyzed for baseline comparisons starting month 16 and outcomes months 55 to 60.

**Table 5:** Projected timeline for study aims.

Study task	Study month								
	1-3	4-11	12-19	20-27	28-35	36-43	43-48	49-54	55-60
Staff preparation	X^a^	—^b^	—	—	—	—	—	—	—
Participant recruitment: 70-142 patients per year	—	X	X	X	X	X	X	X	—
DT training for nurses or chaplains (before DT; ongoing for fidelity)	—	—	X	X	X	X	X	X	—
Data Processing	—	X	X	X	X	X	X	X	X
Data Analysis, reports, and manuscripts	—	—	X	—	—	—	—	—	X

^a^X: data collected

^b^–: not applicable.

## Results

Funding was obtained in 2016 with participant enrollment starting in 2017. Results are expected in 2021.

## Discussion

### Potential Problems and Alternate Plans

This rigorous trial of DT will constitute a landmark step in palliative care and spiritual health services research. We designed this study mindful of a number of threats to study validity as we implement the study within the workflow of outpatient palliative care in 6 sites and present plans to achieve study aims.

### Recruitment

Anticipated numbers of potential participants are based on prior studies among similar patient populations both in in-patient and out-patient settings. However, recruitment is always difficult, especially in palliative care settings. We recruited 6 sites to ensure a generous pool of eligible patients and to use a stepped-wedge design for efficiency in the required sample size. Also, the stepped-wedge design allows flexibility in that, if recruitment proves difficult as proposed, we can add steps and sites to ensure a sufficient sample**.**

### Differences Between Sites

We include 6 sites and will recruit participants from outpatient services only. We carefully selected sites with consideration of geographic and population diversity, but our analytic model will include random effect terms to account for site differences, should they occur.

### Attrition

Attrition due to illness burden and death is inevitable in this population. We will monitor attrition carefully throughout the study. If the estimates we have used prove inaccurate we will adjust recruitment numbers accordingly to meet benchmarks throughout the study at each site. We will also analyze data from participants who did not complete the DT to check for differences in patient characteristics and to verify that there is no association between attrition and intervention group assignment.

### Patients Not Returning to Clinic

Including these patients limits potential loss of patients before they can complete the intervention, for instance, during the interval between the first interview and returning the legacy document. Patients’ will be located through the doctor’s office or family; those in the hospital at posttest will finish their intervention at the hospital or by phone with electronic or express mail delivery of the legacy document and will still be included in the study. Including these patients will minimize any potential bias. Furthermore, it ensures that the intervention effect size we estimate will reflect the average effect on a realistic patient population, a portion of which will decline in health status before the intervention is complete.

### Data Integrity

We will collect preintervention data using self-report on a tablet with a smart pen. Participant operation of the device may limit data entry, but we will use Dr. Wilkie’s extensive expertise in using tablets in the homes of elderly hospice patients with cancer. The RA will be present to assist with data collection if needed. We will track incomplete data patterns and raise the topic at regular study meetings to brainstorm solutions such as an improved user interface or directions. We will collect postintervention data by phone or in-person, if the participant has a clinic visit. Obtaining correct phone information in an era when landlines are rare and patients may not be next to their cell phones may be challenging. We will ask for a backup phone number for this reason. Survey responses by phone may be particularly challenging for our sick population; our training will include protocols for taking a break and repeating questions that allow for the illness burden of our participants.

### Availability of Nurses or Chaplains

Each site has a chaplain available to the palliative care service, but others are available to cover for absences (vacation, illness, etc). Similarly, nurses are available. For each site, at least two interventionists will be trained and immediately replaced by another should there be an absence, resignation, or retirement.

### Summary

We have exceptionally strong research and clinical environments to support the proposed study, and a strong, collaborative, interdisciplinary team distinguished by its rare combination of chaplaincy, nursing, and palliative care researchers. We are noted for a history of excellence in palliative care research in elderly cancer patients, including a Templeton Foundation grant to stimulate chaplaincy research in palliative care and several funded R01 level studies focused on palliative care populations. We propose a highly significant and high-quality study in which we will apply rigorous science to an area that sorely needs it: spiritual care research. Studying 560 participants in a pre- and posttest, randomized, controlled 4-step, stepped-wedge design, we will compare the effects of usual outpatient palliative care against the same plus either nurse-led or chaplain-led DT on patient outcomes (dignity impact, existential tasks, and cancer prognosis awareness). Using a multilevel analysis with site, provider (nurse and chaplain), and time (step) in the model, we will determine the efficacy and mechanism of DT when delivered by nurses or chaplains as a spiritual care therapy. Success in this landmark study will yield the first manualized intervention for chaplaincy services, its potential efficacy compared with nurse-led DT, and insights into its mechanisms of action related to spiritual care, an area of great importance to elderly cancer patients receiving palliative care. We will *disseminate* study findings in a variety of venues for presentation and publication to reach palliative care, oncology, gerontology, chaplaincy, nursing, and other audiences.

## Appendix

Multimedia Appendix 1 Untitled.

## Abbreviations

BCC: Board Certified Chaplain
Co-I: coinvestigators
DT: dignity therapy
EHR: electronic health record
ESAS-r: Edmonton Symptom Assessment Scale
ICC: intraclass correlation
MMSE: Mini Mental Status Exam
MPI: multiple primary investigators
NCI: National Cancer Institute
NIH: National Institutes of Health
NINR: National Institute of Nursing Research
PDI: Patient dignity inventory
PPS: Palliative Performance Scale
PSI-C: Patient Satisfaction Instrument-Chaplaincy
RA: research assistants
RCT: randomized controlled trial
