# Supplementary material for: Dignity Therapy Led by Nurses or Chaplains for Elderly Cancer Palliative Care Outpatients: Protocol for a Randomized Controlled Trial
Source: JMIR Res Protoc. 2019 Apr 17;8(4):e12213. doi: 10.2196/12213 (PMC6492061; doi:10.2196/12213)
Supplement: Multimedia Appendix 1 [file resprot_v8i4e12213_app1.pdf]

SUMMARY STATEMENT
(Privileged Communication)

PROGRAM CONTACT:
Ann O'Mara
(240) 276-7074
ao45s@nih.gov

Release Date: 11/05/2015

Application Number: 1 R01 CA200867-01A1

Principal Investigators (Listed Alphabetically):
EMANUEL, LINDA LEAH MD, PHD
FITCHETT, GEORGE PHD
WILKIE, DIANA J PHD (Contact)

Applicant Organization: UNIVERSITY OF FLORIDA

Review Group: ZCA1 RPRB-M (J1)
National Cancer Institute Special Emphasis Panel
NCI R01 Review

Meeting Date: 10/23/2015
Council: JAN 2016
Requested Start: 04/01/2016

RFA/PA: PA13-354
PCC: 3XCM
Dual IC(s): NR, AG

Project Title: Dignity Therapy RCT led by Nurses or Chaplains for Elderly Cancer Outpatients

SRG Action: Impact Score: 28 Percentile: 13 #
Next Steps: Visit http://grants.nih.gov/grants/next_steps.htm
Human Subjects: 30-Human subjects involved - Certified, no SRG concerns
Animal Subjects: 10-No live vertebrate animals involved for competing appl.
Gender: 1A-Both genders, scientifically acceptable
Minority: 1A-Minorities and non-minorities, scientifically acceptable
Children: 3A-No children included, scientifically acceptable
Clinical Research - not NIH-defined Phase III Trial

Project Year	Direct Costs Requested	Estimated Total Cost
1	499,995	776,097
2	499,998	776,101
3	499,998	776,101
4	499,995	776,097
5	499,996	776,098
TOTAL	2,499,982	3,880,495

ADMINISTRATIVE BUDGET NOTE: The budget shown is the requested budget and has not been adjusted to reflect any recommendations made by reviewers. If an award is planned, the costs will be calculated by Institute grants management staff based on the recommendations outlined below in the COMMITTEE BUDGET RECOMMENDATIONS section.

RESUME AND SUMMARY OF DISCUSSION: The goal of this project is to improve psychosocial and spiritual care outcomes for elderly patients with cancer by optimizing dignity therapy interventions. This is an important and understudied area of research that could improve quality-of-life in this patient population, which would be highly significant. Other strengths include the novel stepped-wedge design of the randomized clinical trial that will maximize what can be learned from a potentially difficult population to study, the outstanding interdisciplinary team of investigators that bring complementary expertise to the project, and use of six different sites from different geographic regions which enhances generalizability. Also, the aims are well described, feasible, and supported by strong preliminary data, and the majority of concerns raised in the prior review of this resubmitted application have been addressed adequately. However, there is remaining uncertainty regarding potential effects of missingness on interpretation of the results, plans for multiplicity testing are not sufficiently discussed, and how variations in the delivery of the intervention will be managed remains unclear. Also, there is uncertainty as to whether patients who do not fall in the low or high end of spiritual distress will be included in the study, and while the revised accrual and withdrawal rates are more realistic, these may still be somewhat optimistic. Nonetheless, strengths outweigh the weaknesses and if successful, this project could lead to enhanced adoption of dignity therapy resulting in improved psychosocial and spiritual well-being in elderly cancer patients, which could have an important impact for this patient population.

DESCRIPTION (provided by applicant): Our long-term goal is to improve spiritual care outcomes for elderly patients facing a cancer diagnosis. We will use a nurse-led or chaplain-led intervention, Dignity Therapy (DT), focused on dignity conservation tasks such as settling relationships, sharing words of love, and preparing for separation by death. These tasks are central needs for elderly patients with cancer, but it is not clear if DT should be led by nurses or chaplains, the two disciplines within palliative care most available to provide DT. We propose a 3-arm, pre/posttest, randomized, controlled 4-step, stepped-wedge design to compare the effects of usual outpatient palliative care and usual outpatient palliative care along with either nurse-led or chaplain-led DT on pilot tested patient outcomes (dignity impact, existential tasks, and cancer prognosis awareness). We will include 560 elderly patients with cancer from 6 outpatient palliative care services across the U.S. We will assign the 6 sites to usual care in the first-step period (12 months), and randomly assign 2 sites per step to begin and continue DT led either by a nurse or a chaplain during each of the next three steps. During usual care steps, 280 patients will complete pretest measures (patient outcomes, processes, covariates [physical symptoms, spiritual distress]), receive usual chaplain care, and 4-6 weeks later will complete posttest measures. During experimental steps as part of routine palliative care, 280 patients will complete pretest measures, receive nurse-led or chaplain-led DT, and 4-6 weeks later will complete posttest measures. Process measures will be completed during all steps. Using mixed level analysis with site, provider (nurse, chaplain), and time (step) included in the model, we will compare the usual care and DT groups for effects on patient outcomes and spiritual care processes and determine the moderating effects of physical symptoms and spiritual distress. Specific aims are to: Aim 1 Compare usual palliative care and usual palliative care with DT (nurse-led, chaplain-led) groups for effects on: a) patient outcomes (dignity impact, existential tasks, and cancer prognosis awareness). We hypothesize that, controlling for pretest scores, each of the DT groups will have higher scores on the dignity impact and existential tasks measures than the usual care group; each of the DT groups will have better peaceful awareness and treatment preference more consistent with their cancer prognosis than the usual care group; and b) processes of delivering palliative spiritual care services (satisfaction, unmet spiritual needs). We hypothesize that the DT groups will show increased patient satisfaction with spiritual care services and fewer unmet spiritual needs compared to the usual care group. Aim 2 Explore the influence of physical symptoms and spiritual distress on the dignity impact and existential tasks effects of usual palliative care and nurse-led or chaplain-led DT. We hypothesize that physical symptoms and spiritual distress will significantly affect intervention effects. This rigorous trial of DT will constitute a landmark step in palliative care and spiritual health services research.

PUBLIC HEALTH RELEVANCE: Our goal is to improve psychosocial and spiritual care outcomes for elderly patients with cancer by optimizing a nurse-led or chaplain-led intervention focused on dignity conservation tasks such as settling relationships, sharing words of love, and preparing a legacy manuscript. This dignity therapy is known to be well accepted by patients in studies, but it is not widely used and it remains unclear how best it can work in real life settings. We propose a rigorous study to evaluate the effects of usual outpatient palliative care compared with added nurse- led or chaplain-led dignity therapy, to assess dignity therapy's impact on patient dignity, preparation for death/life completion tasks, and spiritual well-being; rigorous evaluation of dignity therapy in real care settings would constitute a landmark step in health services research for elderly patients with cancer.

CRITIQUES: The written critiques of individual reviewers are provided in essentially unedited form in this section. Please note that these critiques and criterion scores were prepared prior to the meeting and may not have been revised subsequent to the discussions at the review meeting. The "Resume and Summary of Discussion" section summarizes the final opinions of the review committee.

CRITIQUE 1:

Significance:	2
Investigator(s):	1
Innovation:	2
Approach:	3
Environment:	1

Overall Impact: The proposed study is a randomized trial of usual outpatient palliative care vs usual outpatient palliative care with either nurse-led or chaplain-led dignity therapy on patient outcomes (Primary: dignity impact; Secondary: existential tasks and cancer prognosis awareness). This is an important yet understudied area of gero-oncology palliative care – this study will advance the field through a rigorous test of a manualized program. Engagement of Dr. Chochinov [developer of the intervention] is an important strength. The research team is interdisciplinary with investigators who have worked together productively. While there are challenges in conducting a study across six sites, the team has the experience and expertise to do so successfully. The approach is well thought out and uses a novel step-wedge design to maximize what can be learned from a potentially difficult population to study. Many of the issues raised in the prior review have been clarified in this resubmission. A couple of minor weaknesses remain in terms of the approach but are easily addressed and do not significantly dampen enthusiasm for the project. Potential impact, overall, is expected to be high.

1. Significance:
Strengths
- The proposed study addresses a much understudied area – spiritual care interventions for elderly cancer patients – using Dignity Therapy as the intervention. There is a major gap in the area of palliative care interventions, especially spiritual care interventions, for this population.
- The findings from the proposed study will advance the field of gero-oncology palliative care by providing a manualized intervention that could be widely translated into clinical practice settings.

Weaknesses
- None noted.

2. Investigator(s):
Strengths
- The Multiple Principal Investigator leadership team is an outstanding interdisciplinary group, and the plan is clearly articulated.

- Members of the research team have a demonstrated history of collaboration including joint publications.
- Dr. Chochinov, who developed the Dignity Therapy intervention program, including the manual, is a consultant on the project. This is an important strength of the research team.

Weaknesses

- None noted.

3. Innovation:

Strengths

- Use of the stepped wedge design, which requires few participants, is innovative.
- Framing the Dignity Therapy intervention as a spiritual intervention and focusing on spiritual outcomes as a primary outcome is innovative.

Weaknesses

- None noted.

4. Approach:

Strengths

- Clearly described conceptual framework that is linked to the aims and to the overall study design.
- Well written description of the clinical trial of the intervention.
- Quota sampling with 50% demonstrating 'low' spiritual distress and 50% demonstrating 'high' distress at each site/step ensures variability.
- Use of six different sites from different geographic regions enhances generalizability.
- The intervention is manualized and has demonstrated feasibility.
- Plan to explore potential moderators as a precursor to future studies is a strength.

Weaknesses

- Unclear if those who fall somewhere between 'low' and 'high' in spiritual distress will be included; if excluded, there needs to be a clear rationale for not including those persons, especially in a potentially difficult group to engage in the study.
- Still not clear how variations in the type of usual palliative care that is delivered in each of the six sites will be documented.

5. Environment:

Strengths

- Primary sites which will support the multi-site coordination efforts are outstanding.
- The six different clinical sites from different geographic regions are all excellent in terms of the clinical and research resources to support the project.

Weaknesses

- None noted.

Protections for Human Subjects: Acceptable Risks and/or Adequate Protections

- Risks described with appropriate plans to protect against risks

Data and Safety Monitoring Plan (Applicable for Clinical Trials Only): Acceptable

  - Plans for monitoring described.

Inclusion of Women, Minorities and Children and not IRB Exemption #4.

- Sex/Gender: Distribution justified scientifically
- Race/Ethnicity: Distribution justified scientifically
- Inclusion/Exclusion of Children under 21: Excluding ages < 21 justified scientifically
- Specific plans for recruiting minorities are described.

Vertebrate Animals: Not Applicable (No Vertebrate Animals).

Biohazards: Not Applicable (No Biohazards).

Resubmission:
- Very responsive to prior critiques.

Resource Sharing Plans: Acceptable.

Budget and Period of Support: Recommend as requested.

CRITIQUE 2:

Significance:	3
Investigator(s):	1
Innovation:	3
Approach:	3
Environment:	2

Overall Impact: This resubmitted application describes a 3-arm, pre/post-test, randomized controlled 4-step wedge designed efficacy trial that will compare the effects of usual outpatient palliative care and usual outpatient palliative care with either nurse-led or chaplain-led Dignity Therapy (DT) on pilot-tested dignity impact, existential tasks, and cancer prognosis awareness. Concerns raised in the prior review of this application are generally addressed. Strengths of the application include the innovation, investigators, and environments. Some of the weaknesses identified in the prior review of the application remain of concern, such as whether provider delivery of dignity therapy is the main issue, especially given the lack of availability of board certified chaplains. Also, whether the outcome measures of Aim 1 will be adequately sensitive to the intervention's effect remains unclear. There is some concern that more preliminary data may be needed before conducting this large, multi-site randomized controlled trial. Overall, if successful, the project is expected to have a moderately high impact.

1. Significance:
Strengths
- Application addresses the need to advance the understudied scientific area of geriatric palliative care for cancer patients.
- The study focuses on a spiritual intervention.
- The study includes older adults in the home setting, not restricted to hospice settings.

Weaknesses
- There is uncertainty as to whether Aim 1 outcome measures will be adequately sensitive to the intervention's effect.

2. Investigator(s):
Strengths
- Principle Investigator has extensive knowledge and experience in conducting RCTs.
- The Principal Investigator has assembled an excellent team and six site directors to conduct this study; many have worked together on research and publications.
- Dr. Chochinov, developer of the Dignity Therapy program, is a consultant on the study, which is an important strength.

Weaknesses

- None noted.

3. Innovation:
Strengths
- A rigorous trial of Dignity Therapy will be a landmark step in palliative care and spiritual health services research.
- Use of the stepped-wedge design has advantages for recruitment and smaller sample size in a palliative care context.
- Application proposes a novel method to impact spiritual care in a 4-6 week period.

Weaknesses
- There is minor concern regarding capacity issues for delivery of this intervention by nurses and board-certified chaplains.

4. Approach:
Strengths
- The approach section has been modified in response to several concerns raised in the prior review of this application.
- Use of a manualized nurse-led or chaplain-led intervention to insure rigor at each site and with numerous research team members is a strength.

Weaknesses
- The issue of sample variability on spiritual distress and how it impacts delivery of Dignity Therapy intervention is not sufficiently addressed.
- The revised accrual and withdrawal rates are more realistic but there is some concern that both estimates remain somewhat optimistic.
- There is remaining concern that additional preliminary data may be needed.

5. Environment:
Strengths
- The team has a history of excellence in palliative care research in elderly cancer patients.
- The study will use six strong research and clinical environments to conduct the study.

Weaknesses
- Issue of coordination is a concern among sites where investigators are simultaneously recruiting palliative care patients for other studies.

Protections for Human Subjects: Acceptable Risks and/or Adequate Protections
Data and Safety Monitoring Plan (Applicable for Clinical Trials Only): Acceptable

Inclusion of Women, Minorities and Children and not IRB Exemption #4.
- Sex/Gender: Distribution justified scientifically
- Race/Ethnicity: Distribution justified scientifically
- Inclusion/Exclusion of Children under 21: Excluding ages < 21 justified scientifically

Vertebrate Animals: Not Applicable (No Vertebrate Animals).

Biohazards: Not Applicable (No Biohazards).

Resubmission: Responsive to many of the prior concerns.

Resource Sharing Plans: Acceptable.

Budget and Period of Support: Recommend as requested.

CRITIQUE 3:

Significance:	3
Investigator(s):	2
Innovation:	2
Approach:	4
Environment:	3

Overall Impact: The plan is to study an intervention delivered by nurses or chaplains to address patients' spiritual needs at end of life. The intervention is well-described, and, if successful, would have the potential to ameliorate suffering of both the patients and their survivors. The investigators are experienced and have worked together, and present preliminary data that the intervention is feasible and could have a positive effect on patients' spiritual outcomes. Studying patients' spiritual endpoints as a primary outcome in and of itself is quite innovative. The design of the study is well thought out. However, potential impact is somewhat tempered by concerns about the complexity of the study, the multiplicity of endpoints, the potential effect of informative missingness on the results, and variation in the delivery of the intervention.

1. Significance:
Strengths

- A systematic application of an intervention specifically designed to address patients' spiritual needs at end-of-life could have a significant positive effect both on those patients and on their survivors.

Weaknesses

- The design of the experiment is complex, there is going to be significant variation in the delivery of the intervention both between and within sites and a large number of endpoints will be assessed. There is a concern that the results will therefore be a mixture of a large number of positive and negative results and the overall conclusion will be that more research is needed.

2. Investigator(s):
Strengths

- The Principal Investigator is highly experienced and well qualified to conduct the study. She is the Principal Investigator of an active PCORI grant and a recently completed R01.
- The research team includes experienced practitioners in palliative and end-of-life care, ministry, epidemiology, and psychiatry.

Weaknesses

- There is minor concern regarding the minimal publication and/or grant support record of some of the co-investigators.

3. Innovation:
Strengths

- The use of Dignity Therapy and its analysis via a sophisticated RCT is quite innovative.
- Employing spiritual outcomes ("endpoints" may be more appropriate) as assessments of the effect of DT is novel.

Weaknesses

- None noted.

4. Approach:

Strengths

- The step-wedge design is appropriate to the study.
- The power analysis is not overwhelmingly rigorous, but acceptable. There will almost certainly be inadequate power to compare nurse- and chaplain-led interventions, but that comparison should not be a focus of the study.

Weaknesses

- The effect of the exclusion criteria on the analysis is unclear, as they are complicated and appear related to the study endpoints.
- There is concern that power analyses related to effect sizes, when the meaning of the effect size is unclear, are of little value.
- The nature of missingness could have a profound effect on the interpretation of the results from Aim 1, but this is not sufficiently addressed.
- There is insufficient assessment of the quality of the intervention in the planned analysis of the results. Patients will be clustered not only within sites but within nurses and chaplains within sites, but the analysis model does not adequately deal with this.
- There are a great many endpoints being studied and plans for multiplicity of testing are not adequately presented.
- Aim 2 analysis plan is superficial.
- It is unclear whether the effect on survivors will be adequately studied.

5. Environment:
Strengths

- The study is being conducted at major research institutions that should have the patient resources to recruit to the study.
- Conducting an efficacy, as opposed to an effectiveness study, is not a concern.

Weaknesses

- The Environment section does not adequately describe the data collection and management systems to be implemented for this complex, multi-site study.

Protections for Human Subjects: Acceptable Risks and/or Adequate Protections

- A reasonable analysis of the risk/benefit ratio is presented.

Data and Safety Monitoring Plan (Applicable for Clinical Trials Only): Acceptable

  - Risk is low and an internal DSMB is appropriate.

Inclusion of Women, Minorities and Children and not IRB Exemption #4.

- Sex/Gender: Distribution justified scientifically
- Race/Ethnicity: Distribution justified scientifically
- Inclusion/Exclusion of Children under 21: Excluding ages < 21 justified scientifically

Vertebrate Animals: Not Applicable (No Vertebrate Animals).

Biohazards: Not Applicable (No Biohazards).

Resubmission: Responsive to prior concerns.

Resource Sharing Plans: Acceptable.

Budget and Period of Support: Recommend as requested.

THE FOLLOWING SECTIONS WERE PREPARED BY THE SCIENTIFIC REVIEW OFFICER TO SUMMARIZE THE OUTCOME OF DISCUSSIONS OF THE REVIEW COMMITTEE, OR REVIEWERS' WRITTEN CRITIQUES, ON THE FOLLOWING ISSUES:

PROTECTION OF HUMAN SUBJECTS (Resume): ACCEPTABLE. Potential risks to participants and protections from those risks are addressed adequately. Informed consent will be obtained prior to participation, and procedures are in place to protect patient confidentiality.

INCLUSION OF WOMEN PLAN (Resume): ACCEPTABLE. Both women and men will be included in this study. G1A

INCLUSION OF MINORITIES PLAN (Resume): ACCEPTABLE. All eligible patients will be recruited regardless of racial or ethnic backgrounds. M1A

INCLUSION OF CHILDREN PLAN (Resume): ACCEPTABLE. Children will not be included in this study. C3A

COMMITTEE BUDGET RECOMMENDATIONS: The budget is recommended as requested.

Ad hoc or special section application percentiled against "Total CSR" base.

NIH has modified its policy regarding the receipt of resubmissions (amended applications). See Guide Notice NOT-OD-14-074 at http://grants.nih.gov/grants/guide/notice-files/NOT-OD-14-074.html. The impact/priority score is calculated after discussion of an application by averaging the overall scores (1-9) given by all voting reviewers on the committee and multiplying by 10. The criterion scores are submitted prior to the meeting by the individual reviewers assigned to an application, and are not discussed specifically at the review meeting or calculated into the overall impact score. Some applications also receive a percentile ranking. For details on the review process, see http://grants.nih.gov/grants/peer_review_process.htm#scoring.

MEETING ROSTER

National Cancer Institute Special Emphasis Panel
NATIONAL CANCER INSTITUTE
NCI R01 Review
ZCA1 RPRB-M (J1) S
October 23, 2015

CHAIRPERSON

KUMAR, NAGI B., PHD
SENIOR MEMBER, POPULATION SCIENCES DIVISION
AND DIRECTOR, CANCER CHEMOPREVENTION
DEPARTMENT OF CANCER EPIDEMIOLOGY
DIVISION OF POPULATION SCIENCES
H. LEE MOFFITT CANCER CENTER & RESEARCH INSTITUTE
TAMPA, FL 33612

MEMBERS

ABDI, SALAHADIN , MD, PHD
PROFESSOR AND CHAIR
DEPARTMENT OF PAIN MEDICINE
THE UNIVERSITY OF TEXAS MD ANDERSON CANCER CENTER
HOUSTON, TX 77030

ALIZAD, AZRA , MD
PROFESSOR
DEPARTMENT OF PHYSIOLOGY
AND BIOMEDICAL ENGINEERING
MAYO CLINIC
ROCHESTER, MN 55905

BERGER, ANN M., MSN, PHD, FAAN
FLORENCE NIEDFELT PROFESSOR AND
DOROTHY HODGES OLSON ENDOWED CHAIR IN NURSING
ADVANCE PRACTICE NURSE, ONCOLOGY
COLLEGE OF NURSING
UNIVERSITY OF NEBRASKA MEDICAL CENTER
OMAHA, NE 681985330

DWYER, KATHLEEN A., RN, PHD
PROFESSOR
COLLEGE OF NURSING/CENTER FOR NURSING RESEARCH
THE UNIVERSITY OF OKLAHOMA HEALTH SCIENCES CENTER
OKLAHOMA CITY, OK 73117

HOULETTE, JUDY KASEY, MA
EXECUTIVE DIRECTOR
FRIEND FOR LIFE CANCER SUPPORT NETWORK
LOUISVILLE, KY 40207

IGLEHART, JAMES D., MD
PROFESSOR
DEPARTMENT OF CANCER BIOLOGY
HARVARD MEDICAL SCHOOL
DANA-FARBER CANCER INSTITUTE
BOSTON, MA 02115

NORMOLLE, DANIEL P., PHD
ASSOCIATE PROFESSOR
DEPARTMENT OF BIOSTATISTICS
THE UNIVERSITY OF PITTSBURGH CANCER INSTITUTE
PITTSSBURGH, PA 15213

SLOVIN, SUSAN FAITH, MD, PHD
ATTENDING PHYSICIAN
GENITOURINARY ONCOLOGY SERVICE
MEMORIAL SLOAN-KETTERING CANCER CENTER
PROFESSOR, DEPARTMENT OF MEDICINE
WEILL-CORNELL MEDICAL COLLEGE
NEW YORK, NY 10065

SCIENTIFIC REVIEW OFFICER

LYMAN, CARON A, PHD
CHIEF, SCIENTIFIC REVIEW OFFICER
RESEARCH PROGRAMS REVIEW BRANCH
DIVISION OF EXTRAMURAL ACTIVITIES
NATIONAL CANCER INSTITUTE
NATIONAL INSTITUTES OF HEALTH
BETHESDA, MD 208929750

EXTRAMURAL SUPPORT ASSISTANT

HARLEY, SHANNON
STAFF ASSISTANT
RESEARCH PROGRAM REVIEW BRANCH
DIVISION OF EXTRAMURAL ACTIVITIES
NATIONAL CANCER INSTITUTE
NATIONAL INSTITUTE OF HEALTH
BETHESDA, MD 20850

Consultants are required to absent themselves from the room during the review of any application if their presence would constitute or appear to constitute a conflict of interest.
